# Automated Diagnosis for Colon Cancer Diseases Using Stacking Transformer Models and Explainable Artificial Intelligence

**DOI:** 10.3390/diagnostics13182939

**Published:** 2023-09-13

**Authors:** Lubna Abdelkareim Gabralla, Ali Mohamed Hussien, Abdulaziz AlMohimeed, Hager Saleh, Deema Mohammed Alsekait, Shaker El-Sappagh, Abdelmgeid A. Ali, Moatamad Refaat Hassan

**Affiliations:** 1 Department of Computer Science and Information Technology, Applied College, Princess Nourah Bint Abdulrahman University, P.O. Box 84428, Riyadh 11671, Saudi Arabia; 2 Department of Computer Science, Faculty of Science, Aswan University, Aswan 81528, Egypt; 3 College of Computer and Information Sciences, Imam Mohammad Ibn Saud Islamic University (IMSIU), Riyadh 13318, Saudi Arabia; 4 Faculty of Computers and Artificial Intelligence, South Valley University, Hurghada 84511, Egypt; 5 Faculty of Computer Science and Engineering, Galala University, Suez 34511, Egypt; 6 Information Systems Department, Faculty of Computers and Artificial Intelligence, Benha University, Banha 13518, Egypt; 7 Faculty of Computers and Information, Minia University, Minia 61519, Egypt

**Keywords:** colon cancer, stacking ensemble, CNN, transfer learning, explainable AI (XAI)

## Abstract

Colon cancer is the third most common cancer type worldwide in 2020, almost two million cases were diagnosed. As a result, providing new, highly accurate techniques in detecting colon cancer leads to early and successful treatment of this disease. This paper aims to propose a heterogenic stacking deep learning model to predict colon cancer. Stacking deep learning is integrated with pretrained convolutional neural network (CNN) models with a metalearner to enhance colon cancer prediction performance. The proposed model is compared with VGG16, InceptionV3, Resnet50, and DenseNet121 using different evaluation metrics. Furthermore, the proposed models are evaluated using the LC25000 and WCE binary and muticlassified colon cancer image datasets. The results show that the stacking models recorded the highest performance for the two datasets. For the LC25000 dataset, the stacked model recorded the highest performance accuracy, recall, precision, and F1 score (100). For the WCE colon image dataset, the stacked model recorded the highest performance accuracy, recall, precision, and F1 score (98). Stacking-SVM achieved the highest performed compared to existing models (VGG16, InceptionV3, Resnet50, and DenseNet121) because it combines the output of multiple single models and trains and evaluates a metalearner using the output to produce better predictive results than any single model. Black-box deep learning models are represented using explainable AI (XAI).

## 1. Introduction

Colorectal cancer (CRC) is a type of cancer that affects the large intestine, commonly called the colon. It begins when cells in the colon have mutations in their DNA, causing them to grow and divide uncontrollably. If left untreated, these abnormal cells can become tumors that invade neighboring tissues or spread to other body parts [1]. Colorectal cancer risk factors include age, family history of colorectal polyps or malignancies, inflammatory bowel disease (IBD), smoking, and obesity [2]. Colorectal cancer (CRC) is a prevalent disease that threatens public health, as it affects many people globally [3]. Globally, it ranked third in terms of prevalence and second in terms of death rate [4].

In 2019, 142,462 instances of colon and rectal cancer were reported, with 75,581 males and 66,881 females affected in the United States [5]. In 2023, 153,020 adults will be diagnosed with colorectal cancer [6]. The main risk factors for colon cancer incidence are unhealthy behaviors, such as excessive alcohol use, obesity, smoking, a diet high in red and processed meat, advanced age, and family history of the disease [6]. Consequently, there is a constant need for a highly accurate system for detecting colon cancer at a very early stage, which can lead to prevention of the disease’s development, a reduction in the associated risks, and support for early treatment.

Deep learning has made essential contributions to the healthcare field by allowing for the development of powerful algorithms capable of analyzing medical data, making predictions, and assisting in various medical tasks such as medical imaging analysis and illness detection [7,8,9,10]. Deep learning techniques are more accurate in image analysis than other conventional ML techniques and traditional methods such as colonoscopy [11], histopathology [12], and functional tomography (PET-CT) [13] because they are able to learn the deep spatial representations from images, improve the quality of results, and increased efficiency. Deep learning is also faster than traditional methods in detecting cancer [14,15,16].

A convolutional neural network (CNN) is a form of deep learning (DL) algorithm frequently utilized for text mining [17], as well as image [18] and video recognition tasks [19]. CNNs automatically learn and extract increasingly complex features from input images or videos by employing convolutional and pooling layers to create higher-level input representations. Furthermore, CNNs can learn autonomously, enabling them to identify intricate visual patterns without relying on human-designed features [20,21]. CNN is the most effective framework for detecting and classifying medical images, as it can identify image patterns and extract essential features from them [22]. Data augmentation techniques such as cropping, flipping, rotation, etc., are important to enhance the effectiveness and results of CNN models [23]. Pretrained CNN models such as VGG16, InceptionV3, DenseNet121, and ResNet50 are used mostly to solve complex problems in image processing and computer vision [24,25]. For example, Babu, Tina, et al. extracted features using pretrained CNN models (Alexnet, VGG-16, and Inception-V3) and used extracted features to train SVM to classify colon cancer images [24]. Garg and Somya utilized pretrained CNN-based models to identify colon cancer with augmentation techniques [25].

Ensemble learning is a powerful machine learning (ML) [26] technique that combines multiple models to create more accurate, robust, and reliable predictions. By combining different models, ensemble methods can reduce the variance of individual predictors while also improving accuracy [27]. Ensemble techniques can also help improve generalization performance by reducing overfitting caused by single-model approaches [27]. There are various types of ensemble learning, including bagging, boosting, stacking, and voting [28]. For example, Sharma et al. applied voting ensemble learning based on CNN models with the Xception and ResNet models. Younas et al. proposed a weighted ensemble model by combining six CNN models [29].

The main contributions of this paper are summarized as follows:

- A stacking model is developed based on integration of the output of pretrained base models (VGG16, InceptionV3, DenseNet121, and ResNet50) with a meta-learning (SVM) model to enhance performance;
- Stacking-SVM models are compared with VGG16, InceptionV3, DenseNet121, and ResNet50 using various evaluation methods and two image databases;
- Stacking-SVM achieves the best results compared to other models;
- Black-box deep learning models are represented using explainable AI (XAI).

The rest of this paper is organized as follows. Section 2 discusses colon-cancer-related work and briefly describes related literature. Section 3 discusses the architecture of the proposed system to predict colon cancer. Section 4 provides a discussion and analysis of the results. Finally, the paper is concluded in Section 5.

## 2. Related Work

In previous studies, a wide range of ML approaches have been proposed for the analysis of CRC. Most works have used k-means, KNN, and SVM [30,31,32].

CNN has been used successfully to classify colon cancer in recent years. CNNs can extract relevant features from medical imaging data and apply them to the classification task, allowing for a more accurate disease diagnosis. By utilizing CNNs, it is possible to create a DL model that can accurately distinguish between benign and malignant tumors with high accuracy rates. Furthermore, by leveraging transfer learning techniques, such as by fine tuning pretrained models on large datasets of labeled images, researchers have achieved even higher performance levels when classifying colon cancer using CNNs [29]. For example, in [15], the authors applied CNN models (AlexNet, VGG, ResNet, and DenseNet) and inception models to the CRC-5000, AiCOLO, nct-crc-he-100k, and Warwick colon cancer datasets. The results revealed that the ResNet model outperformed other models in terms of accuracy. In [24], the authors extracted features using pretrained CNN models (AlexNet, VGG-16, and Inception-V3) and used extracted features to train an SVM to classify colon cancer. Inception-V3 was found to be the most accurate model using Indian datasets. In [25], the authors utilized pretrained CNN-based models (VGG16, NASNetMobile, InceptionV3, InceptionResNetV2, ResNet50, Xception, MobileNet, and DenseNet169) to identify colon cancer with augmentation techniques using the LC25000 dataset. In [33], the authors proposed an approach based on the integration of different techniques (Modified ResNet-50, principal component analysis (PCA), and AdaBoost) using a combination of three datasets: the Kvasir, ETIS-LaribPolypDB, and CVC-ClinicDB datasetsIn [29], the authors presented a prediction system for classification of colorectal polyps based on the CNN architecture. Multiple pretrained CNN architectures were compared to determine the best hyperparameter settings to improve metric evaluation results. The results revealed that the suggested method achieved a high performance score. In [34], the authors presented a novel context-aware DNN for colon cancer classification using colorectal adenocarcinoma histology images. According to the results, context-aware DNNs performed the best. In [35], the authors presented a colon cancer diagnosis system based on a CNN, supervised learning, and morphological operations. From the results, the proposed method achieved a high accuracy. In [36], the authors suggested a DCNN model for classification of benign and adenocarcinoma colon tissues. They used the LC25000 dataset. The results showed that the proposed approach performed well in classifying assessed cancer tissues. In [37], the authors combined AI algorithms with hyperspectral imaging (HSI) to diagnose colorectal cancer. The authors obtained a dataset from a University in Germany. The HSI with the NN achieved high accuracy. In [38], the authors used six models (LightGBM, SVM, MLP, LDA, XGBoost, and RF) to classify histopathological images using the LC25000 dataset. The experimental results showed that the XGBoost model achieved the best performance. In [16], the authors modified MobileNetV2 and added two layers (max pooling and average pooling layers) to classify colon cancer using the LC25000 dataset. Modified MobileNetV2 achieved the best performance. In [39], the authors proposed a novel DL-based supervised learning model using different augmentation methods on the LC25000 dataset. In [40], the authors used pretrained CNN: models MobileNetV2 and InceptionResnetV2 on the LC25000 dataset. Transfer learning outperformed a fully pretrained CNN, achieving the highest accuracy.

The authors of [41] used the WCE dataset and suggested a novel nested feature fusion method for the fusion of deep features retrieved by the pretrained EfficientNet family to develop a method for the early classification of colorectal cancer. Compared to other models, the proposed method was more accurate. In [42], the authors used pretrained CNN models VGG-16, ResNet-18, and GoogLeNet to detect colon cancer. In comparison with other models, the VGG16 model achieved the best accuracy.

In [43], the authors used two datasets to apply voting ensemble learning based on CNN models: Xception and ResNet. The voting ensemble model recorded the best performance for polyp detection in colonoscopy images, with an acceptable level of all performance measures. In [29], the authors proposed a weighted ensemble model by combining six CNN models using the UCI and PICCOLO datasets. They also used different methods of data augmentation and optimization techniques to ensure the accuracy of the classification model. In [44], the authors proposed StackBox, which combines the prediction outputs from different models (RetinaNet and EfficientDet), with a metalearner using the BKAI-IGH NeoPolyp dataset.

## 3. Methodology

**Figure 1:**
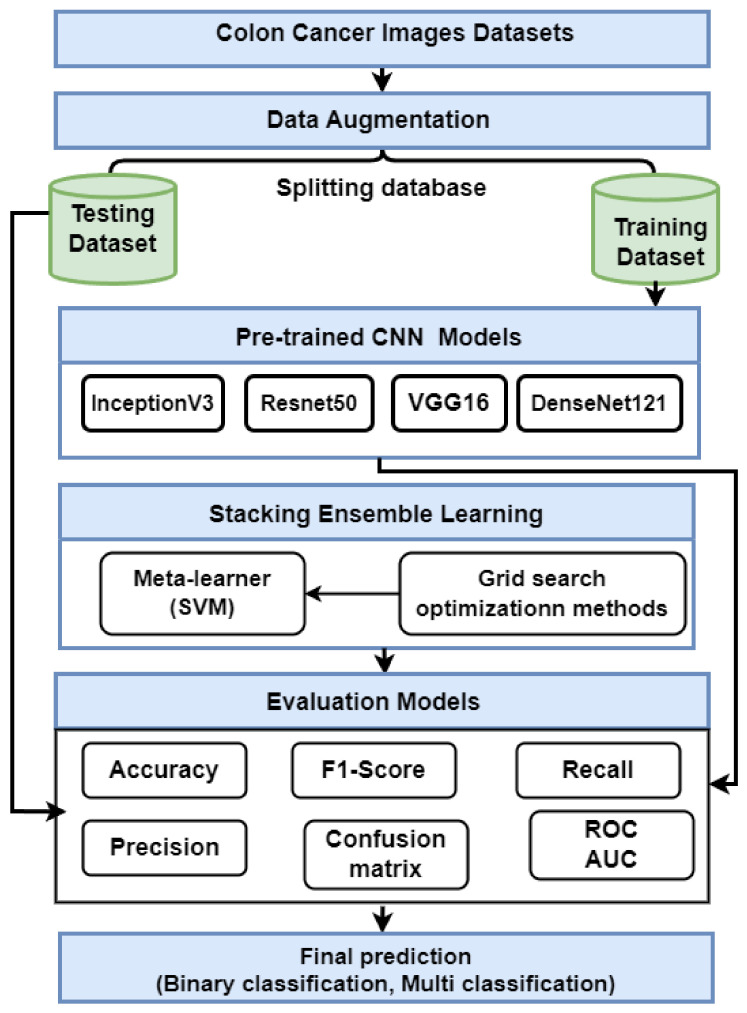
The proposed method for predicting colon cancer.

This section presents the proposed strategy for detecting colon cancer using histopathology images, as shown in Figure 1. The proposed approach includes many steps: data collection; data augmentation; and description Transfer learning using VGG16, ResNet50, InceptionV3, and DensNet121. Finally, we describe an ensemble deep learning model.

### 3.1. Data Collection

We used two colon image datasets for our experiments.

- We used a dataset known as LC25000, which contains histopathological images of colon cancer [45]. There are 5000 images for adenocarcinoma and 5000 images for benign colon cancers in the set. The dataset is split into 70% training (7000 images) and 30% testing (2000 images).
- The WCE colon image dataset collected from Bernal from the Universitat Autonoma de Barcelona [46] includes 6000 images with four classes: normal (N), ulcerative colitis (UC), polyps (P), and esophagitis (E). The dataset is split into 75% training (4500 images) and 25% testing (1200 images).

### 3.2. Data Augmentation

Data augmentation techniques transform an image by mapping the points in the image in a different location or manipulating its intensity levels. As a result of this operation, the existing dataset is modified and subsequently added back into the data pool, increasing the dataset’s size. These techniques improve the trained model’s performance [47,48]. We applied different data augmentation methods: rescale = 1./255, rotation_range = 45, zoom_range = 0.2, width_shift_range = 0.2, height_shift_range = 0.2, horizontal_flip = true, and escale = 1./255.

### 3.3. Pretrained CNN Models

We added three layers before the output layer in each pretrained CNN, a flattening layer, and two fully connected layers. The flattening layer converts the output of the convolution layer into a 1D layer that is used as input to the fully connected layers. The final layer is the output layer, which uses a sigmoid activation function for binary classification and a softmax activation function for multiclassification. Detailed descriptions of the pretrained CNN models are provided for each classifier.

- VGG16 is one of the first CNN models to achieve high accuracy on the ImageNet dataset, which contains over one million images divided into 1000 categories. VGG16 is made up of 16 layers (13 convolutional and 3 fully linked). Convolutional layers are organized into blocks, each with a predetermined number of layers (e.g., two or three) [49].
- InceptionV3 is an image categorization architecture based on CNN. InceptionV3 is made up of several convolutional layers, pooling layers, and fully connected layers. InceptionV3 includes a stack of convolutional layers, a global average pooling layer, numerous fully connected layers, and a Softmax output layer [50].
- Resnet50 comprises 50 convolutional layers and includes residual connections with shortcuts that help the model better manage the vanishing gradient problem and effectively train deeper architectures. The architecture is divided into stages, each containing a sequence of convolutional blocks and identity blocks. Each convolutional block contains three convolution layers, whereas each identity block only has one. The ResNet50 architecture’s last layer is a fully connected layer that performs classification [51].
- DenseNet121 is a CNN architecture consisting of four layers: the input layer, transition layer, dense block, and output layer. The input layer receives an image or data as input. The transition layers consist of multiple convolutional operations, which reduce the size of feature maps before entering densely connected blocks. Each dense block comprises several sets of batch normalization followed by Relu activation and then a series of 3×3 Conv2d with the same padding to preserve spatial resolution between two consecutive stages in the network, which helps to achieve faster convergence when training models on large datasets [52].

### 3.4. The Proposed Stacking Ensemble Model

**Figure 2:**
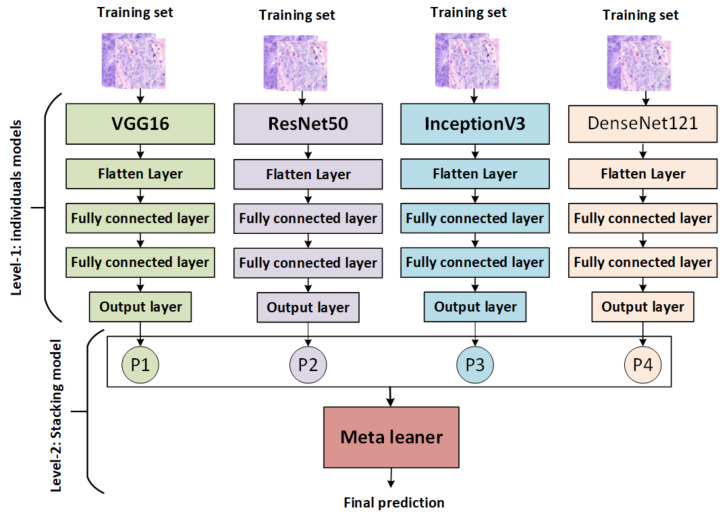
The proposed stacking model.

The stacking ensemble method is a powerful AI model that combines multiple models to produce better predictive results than any single model. It works by training each base model on the same dataset, then combining their predictions via a metamodel that is used to generate more accurate results than traditional methods. It also allows for greater interpretability of the overall result and provides an avenue for further exploration of potential improvements in performance through hyperparameter optimization techniques [53]. There are various types of stacking, including: for example, homogeneous stacking uses base models of the same type [54], whereas heterogeneous stacking uses base models of different types [54]. The proposed stacking ensemble model works in several stages, as shown in Figure 2:

- The pretrained models (VGG16, ResNet50, InceptionV3, and DenseNet121) are trained and saved, then loaded, and all model layers are frozen without the output layers.
- Training stacking combines the output predictions of the training set for each pretrained model. A metalearner (in this case, an SVM) is trained and optimized using stacking. A grid search is used to optimize SVMs as metalearners.
- Testing stacking combines the output predictions of each pretrained model. The metalearner (SVM) is then evaluated using accuracy, precision, recall, F1 score, and ROC analysis.

### 3.5. Evaluating Models

We used different methods to evaluated models:

- Accuracy (ACC), precision (PRE), recall (REC), and F1 score (F1) are the most often-used metrics for classification performance. Equations (1) and (2) illustrate these measures (4).
  True negative (TN) indicates that an individual is healthy and the test is negative, in contrast to true positive (TP), which indicates that the person is ill and the test is positive. When a test shows positive although the subject is healthy, this is known as a false positive (FP). A false negative (FN) occurs when a person is sick but the test is negative
  (1) $$Accuracy=\frac{TP+TN}{TP+FP+TN+FN}.$$
  (2) $$Precision=\frac{TP}{TP+FP}$$
  (3) $$Recall=\frac{TP}{TP+FN}$$
  (4) $$F1-score=\frac{2\cdot precision\cdot recall}{precision+recall}$$
- A confusion matrix (CM) is used to evaluate the performance of models, comprising a table that summarizes an algorithm’s correct and incorrect predictions, with each row representing the actual class and each column representing the anticipated class [55].
- Receiver operating characteristic (ROC) and area under the curve (AUC) are performance metrics for classification problems. ROC represents a probability curve, whereas AUC represents the degree of separability. By indicating the degree of separation between classes, the model is able to perform well. Models with higher AUCs predict better [56].

## 4. Experimental Results

This section describes the experimental setup, as well as the results of CNN models and Stacking-SVM with fixed LR and dynamic LR using two colon image databases.

### 4.1. Experimental Setup

The experiments in this study were implemented using the TensorFlow [57] library, along with Keras [58], both of which were run using the Anaconda-Jupyter notebook platform [59] with an NVIDIA GeForce GT 1030, Intel(R) Core(TM) i5-8500 CPU, and 12.0 GB RAM. For the LC25000 dataset, the number of epochs = 20, activation the function is a sigmoid, the optimizer is Adam, and the loss function is binary_crossentropy, with a fixed learning rate of LR = 0.1. For the WCE dataset, the number of epochs = 50, the activation function is softmax, the optimizer is Adam, and the loss function is categorical_crossentropy, with a fixed learning rate of LR = 0.1.

### 4.2. Performance Analysis of the Pre-Trained CNN and Stacking-SVM Models Using the LC25000 Dataset

All CNN models and Stacking-SVM were applied to the LC25000 dataset for binary classification, in which we distinguished as benign and adenocarcinomas. The LC25000 was split into 70% training set and 30% testing set. The PRE, REC, and F1 for each class were registered; CM and ROC curves are displayed.

#### 4.2.1. Results of Fixed Learning Rate (LR)

Table 1 shows the experimental results for Stacking-SVM and four other CNN models: VGG16, ResNet50, InceptionV3, and DenseNet121 using fixed LR. The Stacking-SVM model had the highest evaluation matrix in terms of PRE, REC, and F1 score average (100).

For the benign class, Stacking-SVM recorded the greatest ACC, PRE, REC, and F1 score (100). DenseNet121 recorded the second-highest results. ResNet50 recorded the lowest performance in REC, REC, and F1 score (85, 65, and 73, respectively). For the adenocarcinomas class, Stacking-SVM registered the highest PRE, REC, and F1 score (100). DenseNet121 had the second-highest results. ResNet50 recorded the lowest performance in terms of PRE, REC, and F1 score (71, 89, and 79, respectively).

**Figure 3:**
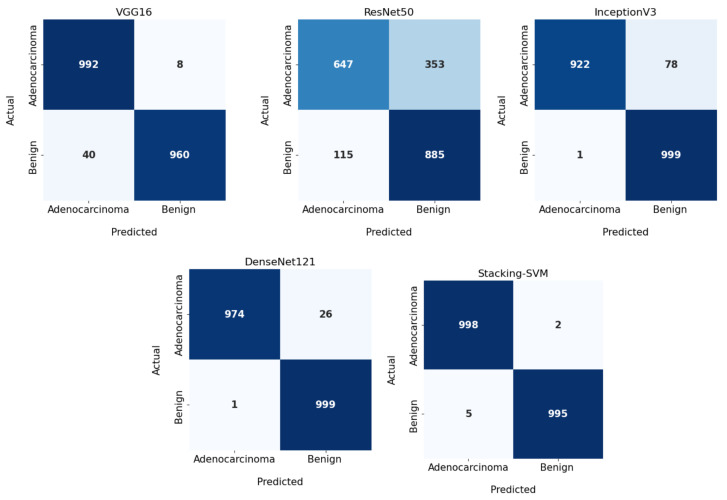
Confusion matrix of pretrained CNN models and the proposed model with a fixed learning rate using the LC25000 dataset.

**Figure 4:**
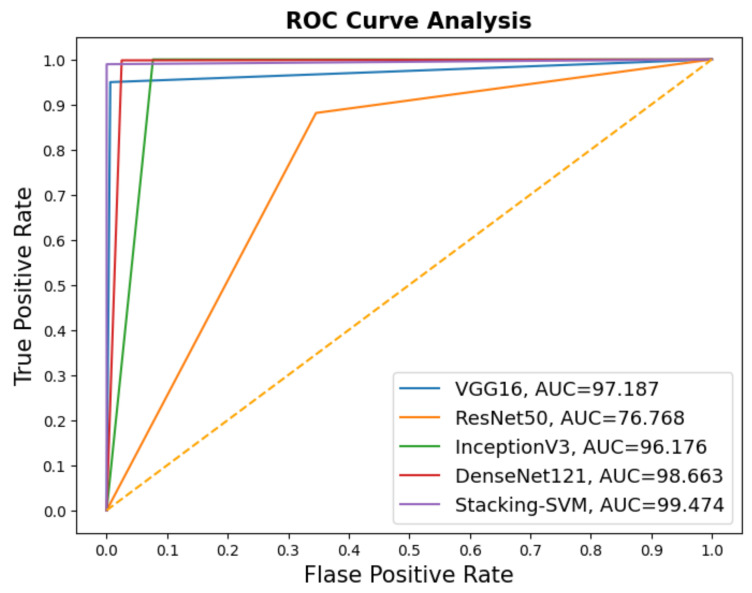
ROC of models with a fixed learning rate using the LC25000 dataset.

In Figure 3 and Figure 4, we show the CMs and ROC curves of the models on the testing set. Using the CM of Stacking-SVM, only 7 of 2000 images were incorrectly classified. When using ResNet50’s CM, 468 of 2000 images were incorrectly classified. The ROC curves are also presented. We can see that Stacking-SVM has the highest AUC, at 99.474, and ResNet50 has the lowest AUC, at 76.768. The Stacking-SVM classifier touches the top-left corner, indicating that it successfully distinguished the samples.

#### 4.2.2. Results of Dynamic Learning Rate (LR)

Table 2 shows the experimental results for Stacking-SVM and four other CNN models: VGG16, ResNet50, InceptionV3, and DenseNet121 using a fixed learning rate. The Stacking-SVM model had the highest F1 evaluation matrix in terms of PRE, REC, and F1 average (98).

For the Benign class, Stacking-SVM recorded the greatest F1, at 98, and DenseNet121 recorded the highest PRE, at 100, with a REC value of 93. ResNet50 recorded the lowest performance in terms of PRE, REC, and F1 score (75, 77, and 76, respectively).

For the adenocarcinoma class, Stacking-SVM registered the highest F1 score, at 98, and VGG16 had the highest PRE, at 100, with a REC value of 87. ResNet50 recorded the lowest performance in terms of PRE, REC, and F1 score (77, 75, and 76, respectively).

**Figure 5:**
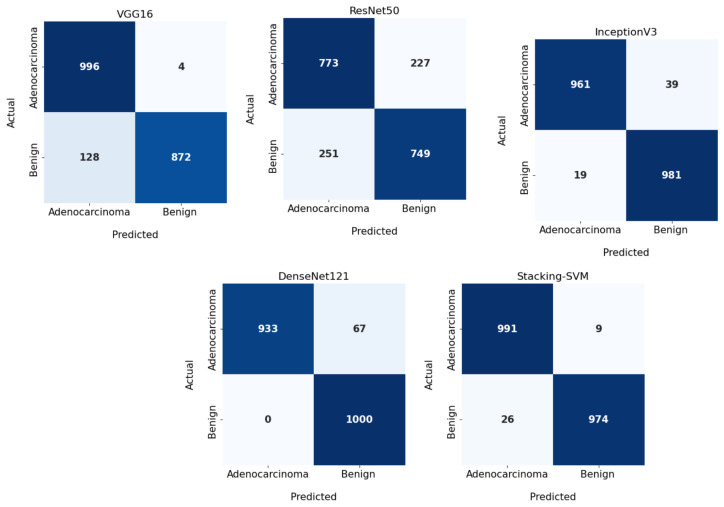
Confusion matrix of pretrained CNN models and the proposed model with dynamic a learning rate using the LC25000 dataset.

**Figure 6:**
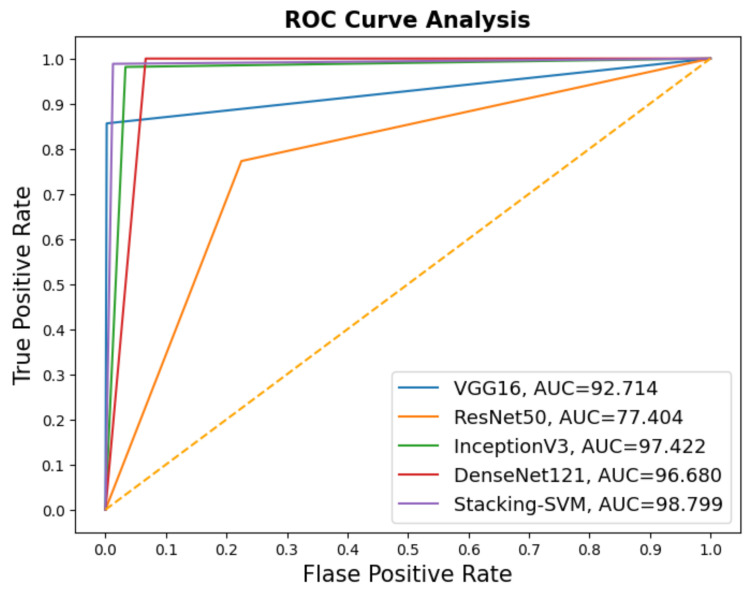
ROC of models with a dynamic learning rate using the LC25000 dataset.

In Figure 5 and Figure 6, we show the CMs and ROC curves of the models on the testing set. Using the CM of Stacking-SVM, only 35 of 2000 images were incorrectly classified. When using ResNet50’s CM, 478 of 2000 images were incorrectly classified. ROC curves are also presented. We can see that Stacking-SVM has the highest AUC, at 98.799, and ResNet50 has the lowest AUC, at 77.404.

### 4.3. Performance Analysis of the Pretrained CNN and Stacking-SVM Models Using the WCE Dataset

All CNN models and Stacking-SVM were applied to the WCE database for multiclassification: normal (N), ulcerative colitis (UC), polyps (P), and esophagitis (E). The dataset was split into 75% training (4500 images) and 25% (1200 images). The PRE, REC, and F1 score for each class were registered; CM and ROC curves are also displayed.

#### 4.3.1. Results of Fixed Learning Rate

Table 3 shows the experimental results of a fixed LR for Stacking-SVM and the CNN models (VGG16, ResNet50, InceptionV3, and DenseNet121) using the WCE dataset. Based on a comparison of other models, the Stacking-SVM model performed best. For the N class, Stacking-SVM recorded the highest PRE, REC, and F1 score (100, 99, and 100, respectively). VGG16 recorded the second-highest results. ResNet50 recorded the lowest performance in terms of PRE, REC, and F1 score (43, 99, and 60, respectively). For the UC class, Stacking-SVM recorded the highest PRE, REC, and F1 score (100, 86, and 92, respectively). VGG16 recorded the second-highest results. ResNet50 recorded the lowest performance in terms of PRE, REC, and F1 (78, 5, and 9, respectively). For the P class, Stacking-SVM recorded the highest PRE, REC, and F1 (88, 100, and 93, respectively). VGG16 recorded the second-highest results. ResNet50 recorded the lowest performance in terms of PRE, REC, and F1 score (73, 11, and 19, respectively). For the E class, Stacking-SVM recorded the highest PRE, REC, and F1 score (99, 100, and 99, respectively). VGG16 recorded the second-highest results. ResNet50 recorded the lowest performance in terms of PRE, REC, and F1 score (65, 96, and 77, respectively).

**Figure 7:**
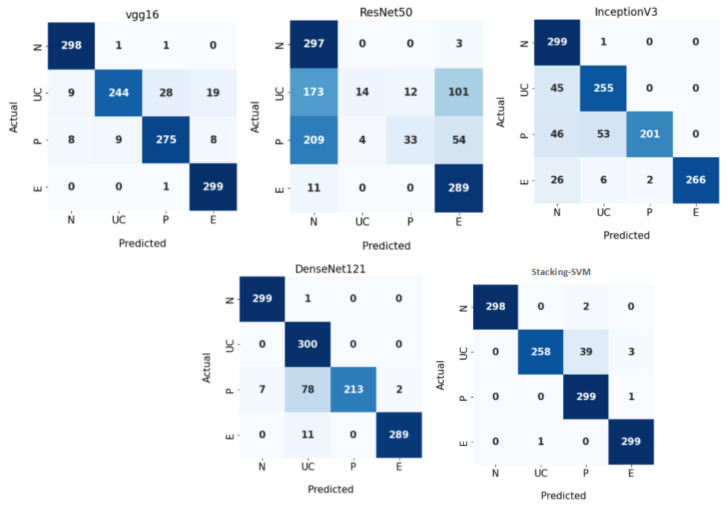
Confusion matrix of pretrained CNN models and the proposed model with a fixed learning rate (LR) using the WCE dataset.

Figure 7 demonstrates CMs for CNN models and Stacking-SVM using a fixed LR on the test dataset. There were four classes of the testing, with 300 images for each class. Stacking-SVM predicted 1154 of 1200 images correctly, with the highest ACC rate of 96.16. VGG16 predicted 1116 of 1200 images correctly, with the second-highest ACC rate of 93. ResNet50 predicted 633 of 1200 images accurately, with the lowest ACC rate of 53.

#### 4.3.2. Results of Dynamic Learning Rate

Table 4 shows the experimental results of dynamic LR for Stacking-SVM and CNN models V (GG16, ResNet50, InceptionV3, and DenseNet121) using the WCE dataset. The Stacking-SVM model had the highest performance compared to other models.

For the N class, Stacking-SVM recorded the highest PRE and F1 score (91 and 93, respectively). VGG16, InceptionV3, and DenseNet121 recorded the highest REC scores, at 100. ResNet50 recorded the lowest performance in terms of PRE, REC, and F1 score (34, 95, and 51, respectively).

For the UC class, Stacking-SVM recorded the highest REC and F1 score (81 and 85, respectively). DenseNet121 recorded the highest PRE, at 99. ResNet50 recorded the lowest performance in terms of PRE, REC, and F1 score.

For the P class, Stacking-SVM recorded the highest REC and F1 score (86 and 87, respectively). DenseNet121 recorded the highest PRE, at 100. ResNet50 recorded the lowest performance in terms of PRE, REC, and F1 score (69, 23, and 34, respectively).

For the E class, Stacking-SVM recorded the highest REC and F1 score (99 and 96, respectively). DenseNet121 recorded the highest PRE, at 100. ResNet50 recorded the lowest performance in terms of REC and F1 score (71 and 77, respectively).

**Figure 8:**
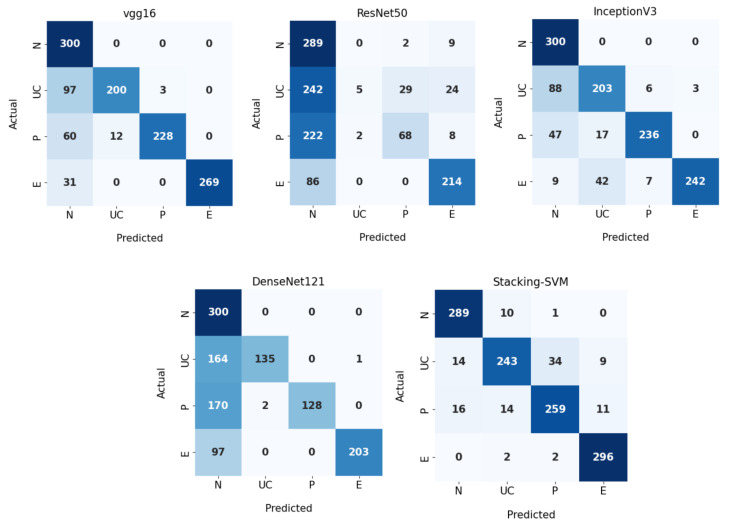
Confusion matrix of pretrained CNN models and the proposed model with a dynamic learning rate using the WCE dataset.

Figure 8 demonstrates CMs for CNN models and Stacking-SVM using a dynamic LR on the test dataset. There were four classes of the testing dataset, with 300 images for each class. Stacking-SVM predicted 1087 of 1200 images correctly, with the highest ACC rate of 91. VGG16 predicted 997 of 1200 images correctly, with the second-highest ACC rate of 84. ResNet50 predicted 576 of 1200 images accurately, with the lowest ACC rate of 48.

### 4.4. Discussion

#### Rate of Model Results with Fixed and Dynamic Learning Rates Using Two Datasets

**Figure 9:**
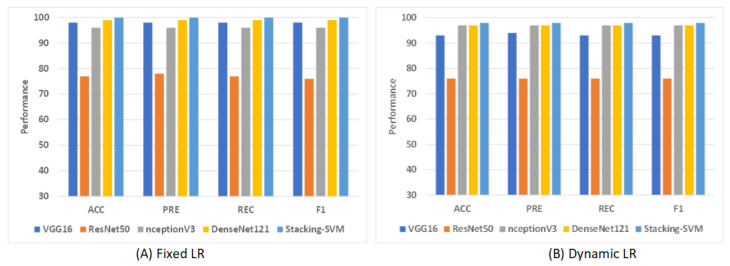
The average rate of model results with fixed and dynamic learning rates using the LC25000 dataset. (**A**) Average rate of model results with a fixed dynamic learning rate; (**B**) average rate of model results with a dynamic learning rate.

Figure 9 shows shows the average rate of model results with fixed and dynamic learning rates using the LC2500 dataset. We can see that the models with a fixed LR achieved the highest results compared to models with a dynamic LR. As shown in Figure 9A, Stacking-SVM recorded the highest average rate, at 100. DenseNet121 recorded the second-highest average rate, at 99, while ResNet50 recorded the worst average rate, at 77. As shown in Figure 9B, Stacking-SVM recorded the highest rate, at 98, and NceptionV3 and DenseNet121 recorded the second-highest average rate, at 97. ResNet50 recorded the worst average rate, at 76.

**Figure 10:**
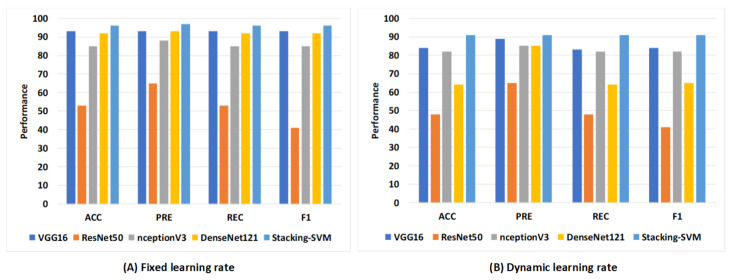
The average rate of model results with fixed and dynamic learning rates using the WCE dataset. (**A**) Average rate of model results with a fixed dynamic learning rate; (**B**) average rate of model results with a dynamic learning rate.

Figure 10 shows the average rate of model results with fixed and dynamic learning rates using the WCE dataset. We can see that the models with a fixed LR achieved the highest results compared to models with a dynamic LR. As shown in Figure 10A, Stacking-SVM recorded the highest average rate of ACC, REC, and F1 score (96), as well as PRE (97). VGG16 recorded the second-highest average rate, at 93, while ResNet50 recorded the worst average rate. As shown in Figure 10B, Stacking-SVM recorded the highest rate, at 91. VGG16 recorded the second-highest average rate, and ResNet50 recorded the worst average rate.

### 4.5. Explainable Artificial Intelligence

**Figure 11:**
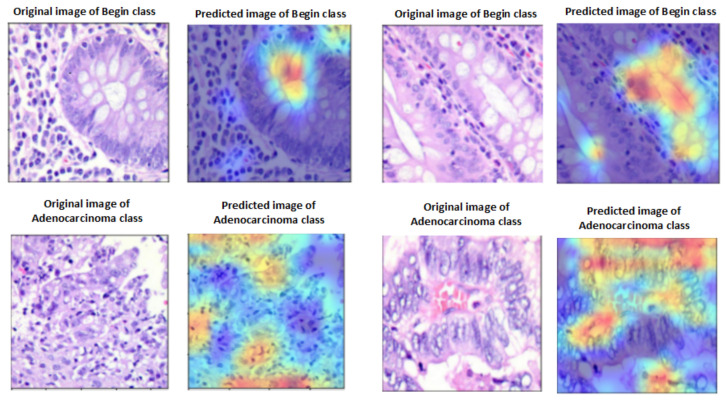
Visualization of deep features for each class for the LC25000 database.

**Figure 12:**
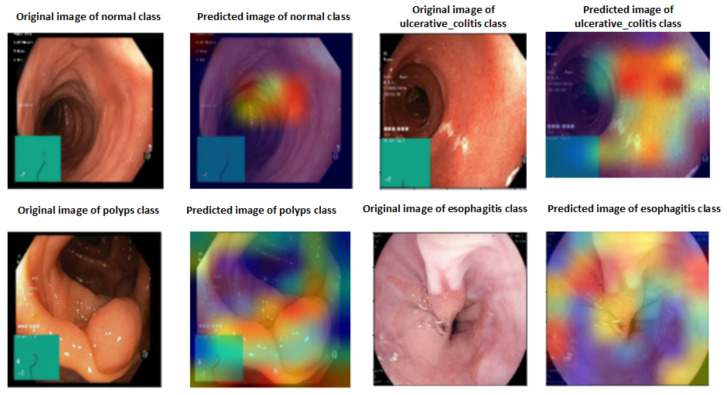
Visualization of deep features for each class for the WCE database.

A heat map of a post hoc explainable model was generated to better understand the behavior of each model. Grad-CAM explainable models were used to extract relevant rich features from the images and generate the heat map for each colon cancer class in the dataset [60]. Grad-CAM maps allow the model to accurately locate textures within an image, thereby improving predictions. The red and yellow areas in the heat map indicate where the CNN model has influenced predictions, while the blue areas are not related to the predictions. Figure 11 shows a visualization of deep features for each class in the LC25000 database. Figure 12 shows the visualization of deep features for each class in the WCE database.

### 4.6. Comparison of Model Results with the Literature

A comparison of the proposed model with other models is shown in Table 5. Based on binary classification using the LC25000 dataset, in [36], the authors used a CNN with PACC = 99.80, REC = 99.87, and F1 = 99.87. In [38], the authors used XGBoost with ACC = 99. In [16], the authors used MobileNetV2 with ACC = 99. In [39,40], the authors used CNN with ACC = 96.33 and 99. In [25], the authors used NASNetMobile with ACC = 98, PRE = 98, REC = 98, and F1 = 98. In [29], the authors applied ensemble learning to classify colon cancer with a private dataset and achieved ACC = 96.3 and PRE = 95.5. Stacking-SVM recorded the highest rate compared to others models, at 100. Based on multiclassification using the WCE dataset, in [41], the authors used EfficientNet with ACC = 94.11. In [42], the authors used VGG16 with ACC = 96.33. In our work, Stacking-SVM recorded the highest performance compared to other models.

## 5. Conclusions

Worldwide, colon cancer ranks third in terms of prevalence; there were almost two million cases diagnosed in 2020. As a result, providing new, highly accurate techniques in detecting colon cancer leads to early and successful treatment of this disease. The main goal of our work was to propose Stacking-SVM based on pretrained CNN models (ResNet50, VGG16, InceptionV3, and DenseNet121) and a metalearner (SVM) to generalize and classify colon cancer using binary classes and multiclasses. The main steps of the proposed framework are data collection, data augmentation, data splitting, model pretraining, and model proposal. In level 1, the output of multiple base models (ResNet50, VGG16, InceptionV3, and DenseNet121) is combined in stacking (training stacking and testing stacking). In level 2, training stacking is used to train a metalearner (SVM). Testing stacking is used to evaluate the metalearner (SVM) and predict the final result. We conducted experiments using two public image databases (LC25000 and WCE) with both fixed and dynamic learning rates. Stacking-SVM models were compared with different pretrained CNN models using other evaluation metrics: ACC, PRE, REC, F1, ROC, AUC, and CMs. The results show that Stacking-SVM with a fixed learning rate achieved the highest average performance for the two databases. The ACC, PRE, REC, and F1 score of the Stacking-SVM model were 100, 100, 100, and 100, respectively, for the LC25000 database. The ACC, PRE, REC, and F1 score of the Stacking-SVM model were 98, 98, 98, and 98, respectively, for the the WCE database. Stacking-SVM recorded the highest performance compared to existing models (VGG16, InceptionV3, Resnet50, and DenseNet121) because it takes the predictions made by multiple single models as inputs, learns to combine them in a way that produces a final prediction, and evaluates the metalearner using the output to produce better predictive results than any single model. In our future work, we plan to aggregate more data to ensure the model’s generalizability. Furthermore, we plan to deploy the developed model in a real clinical system to evaluate its performance on a practical dataset.

## Figures and Tables

**Table 1:** Performance of the five CNN models and Stacking-SVM with a fixed learning rate using the LC25000 dataset.

Model	Class	PRE	REC	F1
VGG16	Benign	96	99	98
	Adenocarcinomas	99	96	98
Average		98	98	98
ResNet50	Benign	85	65	73
	Adenocarcinomas	71	89	79
Average		78	77	76
InceptionV3	Benign	100	92	96
	Adenocarcinomas	93	100	96
Average		96	96	96
DenseNet121	Benign	100	97	99
	Adenocarcinomas	97	100	99
Average		99	99	99
Stacking-SVM	Benign	100	100	100
	Adenocarcinomas	100	100	100
Average		100	100	100

**Table 2:** Performance of the CNN models and Stacking-SVM with a dynamic learning rate using the LC25000 dataset.

Model	Class	PRC	REC	F1
VGG16	Benign	89	100	94
	Adenocarcinomas	100	87	93
Average		94	93	93
ResNet50	Benign	75	77	76
	Adenocarcinomas	77	75	76
Average		76	76	76
InceptionV3	Benign	98	96	97
	Adenocarcinomas	96	98	97
Average		97	97	97
DenseNet121	Benign	100	93	97
	Adenocarcinomas	94	100	97
Average		97	97	97
Stacking-SVM	Benign	97	99	98
	Adenocarcinomas	99	97	98
Average		98	98	98

**Table 3:** Performance of the CNN models and Stacking-SVM with a fixed learning rate (LR) using the LC25000 dataset.

Model	Class	PRE	REC	F1
VGG16	N	95	99	97
	UC	96	81	88
	P	90	92	91
	E	92	100	96
Average		93	93	93
ResNet50	N	43	99	60
	UC	78	05	09
	P	73	11	19
	E	65	96	77
Average		65	53	41
InceptionV3	N	72	100	84
	UC	81	85	83
	P	99	67	80
	E	100	89	94
Average		88	85	85
DenseNet121	N	98	100	99
	UC	77	100	87
	P	100	71	83
	E	99	96	98
Average		93	92	92
Stacking-SVM	N	100	99	100
	UC	100	86	92
	P	88	100	93
	E	99	100	99
Average		97	96	96

**Table 4:** Performance of the CNN models and Stacking-SVM with a dynamic learning rate using the WCE dataset.

Model	Class	PRE	REC	F1
VGG16	N	61	100	76
	UC	94	67	78
	P	99	76	86
	E	100	90	95
Average		89	83	84
ResNet50	N	34	95	51
	UC	71	02	03
	P	69	23	34
	E	84	71	77
Average		65	48	41
InceptionV3	N	68	100	81
	UC	77	68	72
	P	95	79	86
	E	99	81	89
Average		85	82	82
DenseNet121	N	41	100	58
	UC	99	45	62
	P	100	43	60
	E	100	68	81
Average		85	64	65
Stacking-SVM	N	91	96	93
	UC	90	81	85
	P	88	86	87
	E	94	99	96
Average		91	91	91

**Table 5:** Comparison of the proposed model with other models reported in literature.

Ref.	DL Architecture	Dataset(s)	Results (%)
[35]	VGG-16, Resnet-50, SVM	LC25000	ACC = 93
[36]	DeepCNN	LC25000	ACC = 99.80, REC = 99.87, F1 = 99.87
[38]	XGBoost	LC25000	ACC = 99
[16]	MobileNetV2	LC25000	ACC = 99
[39]	CNN	LC25000	ACC = 96.33
[40]	CNN	LC25000	ACC = 99.98
[25]	NASNetMobile	LC25000	ACC = 98, PRE = 98, REC = 98, and F1 = 98
[41]	EfficientNet	WCE dataset	ACC = 94.11
[42]	VGG16	WCE dataset	ACC = 96.33
[29]	the weighted ensemble model	UCI and PICCOLO	ACC = 96.3, PRE = 95.5, REC = 97.2, F1 = 96.3
Our work	Stacking-SVM	LC25000	ACC = 100, PRE = 100, REC = 100, F1 = 100
Our work	Stacking-SVM	WCE	ACC = 98, PRE = 98, REC = 98, F1 = 98

## Data Availability

The direct link in the dataset citations will take you to all of the datasets that were utilized to support the study’s assertions.
